# CD9- and CD81-positive extracellular vesicles provide a marker to monitor glioblastoma cell response to photon-based and proton-based radiotherapy

**DOI:** 10.3389/fonc.2022.947439

**Published:** 2022-09-20

**Authors:** Sara Jennrich, Martin Pelzer, Tobias Tertel, Benjamin Koska, Melanie Vüllings, Basant Kumar Thakur, Verena Jendrossek, Beate Timmermann, Bernd Giebel, Justine Rudner

**Affiliations:** ^1^ Institute of Cell Biology (Cancer Research), University Hospital Essen, University of Duisburg-Essen, Essen, Germany; ^2^ Institute for Transfusion Medicine, University Hospital Essen, University of Duisburg-Essen, Essen, Germany; ^3^ West German Proton Therapy Centre Essen (WPE), West German Cancer Center (WTZ), University Hospital Essen, University of Duisburg-Essen, Essen, Germany; ^4^ Department of Pediatrics III, University Hospital Essen, University of Duisburg-Essen, Essen, Germany

**Keywords:** radiotherapy with photons, radiotherapy with protons, glioblastoma, extracellular vesicles, exosomes, microvesicles, prognostic marker

## Abstract

Glioblastoma multiforme (GBM) is the most aggressive tumor of the central nervous system with a poor prognosis. In the treatment of GBM tumors, radiotherapy plays a major role. Typically, GBM tumors cannot be cured by irradiation because of intrinsic resistance machanisms. An escalation of the irradiation dose in the GBM tumor is difficult due to the high risk of severe side effects in the brain. In the last decade, the development of new irradiation techniques, including proton-based irradiation, promised new chances in the treatment of brain tumors. In contrast to conventional radiotherapy, irradiation with protons allows a dosimetrically more confined dose deposition in the tumor while better sparing the normal tissue surrounding the tumor. A systematic comparison of both irradiation techniques on glioblastoma cells has not been performed so far. Despite the improvements in radiotherapy, it remains challenging to predict the therapeutical response of GBM tumors. Recent publications suggest extracellular vesicles (EVs) as promising markers predicting tumor response. Being part of an ancient intercellular communication system, virtually all cells release specifically composed EVs. The assembly of EVs varies between cell types and depends on environmental parameters. Here, we compared the impact of photon-based with proton-based radiotherapy on cell viability and phenotype of four different glioblastoma cell lines. Furthermore, we characterized EVs released by different glioblastoma cells and correlated released EVs with the cellular response to radiotherapy. Our results demonstrated that glioblastoma cells reacted more sensitive to irradiation with protons than photons, while radiation-induced cell death 72 h after single dose irradiation was independent of the irradiation modality. Moreover, we detected CD9 and CD81-positive EVs in the supernatant of all glioblastoma cells, although at different concentrations. The amount of released CD9 and CD81-positive EVs increased after irradiation when cells became apoptotic. Although secreted EVs of non-irradiated cells were not predictive for radiosensitivity, their increased EV release after irradiation correlated with the cytotoxic response to radiotherapy 72 h after irradiation. Thus, our data suggest a novel application of EVs in the surveillance of anti-cancer therapies.

## Introduction

Glioblastoma multiforme (GBM) is the most common primary central nervous system (CNS)-derived tumor with a poor prognosis, achieving a 5-year-survival rate of only 6.1% (1). Originating from astrocytes and oligodendrocytes, glioblastoma is the most malignant and most aggressive glial cell-derived tumor (WHO grade IV) (2). The current standard of care compromises surgery, followed by radiotherapy and concurrent or adjuvant chemotherapy with the alkylating agent temozolomide (3–5). Thus, radiotherapy constitutes an important treatment option for patients with glioblastoma. Up to 60 Gy total dose in fractions of 1.8 or 2 Gy are routinely administered. Nevertheless, GBM tumors often react refractory to the therapy. Many patients do not tolerate additional treatment with temozolomide very well, and dose escalation with an external beam radiotherapy over 60 Gy (up to 90 Gy) causes an increased risk of severe side effects without improving survival rates (6–8). With increasing irradiation dose over 60 Gy, the risk to damage normal brain tissue rises, thereby impairing neurocognitive function such as memory and speech or facilitating seizures, among others. Despite this treatment intensification, disease progression is still observed in most cases due to tumor recurrence inside and outside the irradiated field. GBM cells become resistant to therapy and invade the surrounding tissue, thus leaving the targeted irradiation field (9–12).

A more confined local dose deposition in the depth of the tissue might spare healthy tissue while allowing dose escalation in the target area containing malignant cells. Among such novel irradiation techniques is proton-based radiotherapy, harboring many advantages over conventional radiotherapy employing X-ray photons (13). The photon-based radiation interacts with the tissue when passing through, thereby depositing energy and damaging the healthy tissue around the tumor by ionization. In contrast, proton-based radiation enters deep into the tissue depositing most of the energy at a well-defined spot in the body when the protons come to rest, sparing completely the tissue behind while losing less energy within the tissue before the tumor. The penetrance depth of proton beams depends on their energy: the higher the energy, the deeper is the tissue penetration. The characteristic increase in energy deposition immediately before protons come to rest in the tissue is called Bragg peak. To adapt the radiation field to the three-dimensional tumor structure, several radiation beams with different energies are superposed, generating a spread-out Bragg peak (SOBP) to enable energy deposition in tissue depth corresponding to the tumor dimension. The linear energy transfer (LET) of protons is lower in shallower tissue depths and rises significantly towards the Bragg peak. Ionization density is therefore lower in the entrance region of tissues and higher in the peak area. Thus, the local energy deposition of highly energetic particles might increase ionizing radiation-induced damage in tumor cells and increase the tumor response to radiotherapy (14). Additional monitoring of the therapy progress might help to adjust the therapy and avoid side effects when GBM cells do not respond to radiotherapy. Monitoring can be achieved using non-invasive techniques such as computer tomography, magnetic resonance tomography or positron-emission tomography. These techniques however require costly equipment and expertly trained staff. Therefore, a quick and cheap detection of therapy response is desirable.

Previously, we demonstrated that glioblastoma cells, like almost all normal non-transformed cells and tumor cells, release extracellular vesicles (EVs) (15). EVs include exosomes derived from the endosomal system (70-150 nm), microvesicles, bud offs from the plasma membrane (100-1,000 nm), apoptotic vesicles that can be as small as exosomes, and apoptotic bodies that can reach sized of up to several micrometers (16, 17). Currently, it is challenging to discriminate exosomes from other EV types of comparable sizes. Accordingly, the International Society of Extracellular Vesicles has recommended collectively deciphering exosome-sized EVs as small EVs (sEVs) (18). EVs were initially considered as part of waste disposal machinery, but later experiments assigned EVs as central mediators of a recently discovered ancient intercellular communication system (19). Independent of their function, EVs are assembled in cell-specific manners as complex aggregates of lipids, proteins and nucleic acids. Detected in all bioliquids, EVs control physiological and pathophysiological processes (20). Due to these properties, EVs emerge as a novel and promising class of biomarkers, which might be exploited to predict the therapeutic outcome in cancer patients.

We recently established imaging flow cytometry (IFCM) analyses as an ideal method for the single EV analysis. The optimization of EV detection procedures enables us, now, to dissect the EV pool in primary body liquids and cell supernatants without the need of preceding purification steps, thus, avoiding potential artefacts eventually arising from the procedures during EV preparation (21–24). Here, we compared the cytotoxic cell response of four different glioblastoma cell lines to photon-based and proton-based radiotherapy in the SOBP’s low LET entrance and the high LET plateau region. To learn whether irradiation of the cells altered the composition of the EV populations in the cell culture supernatants, we labelled EVs with anti-CD9 and anti-CD81 antibodies and analyzed them by IFCM without additional fractionation and purification procedures. Moreover, we intend to correlate the cytotoxic response to radiotherapy with the secretion of sEVs.

## Material and methods

### Cell culture

A172, LN229, U373, and T98G glioblastoma cell lines were purchased from ATCC (Bethesda, MA, USA). According to ATCC, U373 cells used in the present work show genetic similarities to U251 glioblastoma cells. Cells were maintained and grown in a humidified incubator at 37°C and 5% CO2 in RPMI1640 medium supplemented with 10% (v/v) fetal calf serum (Gibco Life Technologies, Eggenstein, Germany). Verification of cell identity was performed using short tandem repeat analysis. Cell morphology was checked weekly using microscopy. Cells were tested for mycoplasma contamination at regular intervals.

### Irradiation

Cells were irradiated in cell culture dishes usually the day after plating. Irradiation with photons was performed with an X-ray machine (Precision X-Ray Inc., North Branford, CT, USA) operated at 320 kV, 12.5 mA with a 1.65-mm Al filter. At a distance of 50 cm from the irradiation source, cells received 3.7 Gy/min.

Proton beam irradiation was carried out at a clinical proton therapy facility employing a 230MeV cyclotron (Proteus Plus, IBA, Louvain-La-Neuve, Belgium) as described previously (14). Cells were positioned on a patient table, aligned with the help of clinical positioning aids, and irradiated in pencil beam mode in a defined source axis distance. Cells were exposed to proton beam in an SOBP’s entrance region (low LET protons) as well as the plateau region (high LET protons). The proton beam was composed of protons of six different energies (in MeV: 110; 107.6; 105.1; 103.1; 100.9; 100). The maximum energy of 110 MeV corresponds to a beam range of approximately 9 cm in water, the lowest energy of 100 MeV to a range of approximately 7.6 cm in water. The high LET proton beam was therefore transmitted through a range shifter (thickness 7.4 cm). To irradiate with the same dose of low LET protons, the range shifter was removed. Irradiation fields were created and optimized by the clinical planning system (RayStation, RaySearch, Stockholm, Sweden) and calibrated by measuring the dose with a 2D array detector MatriXX PT (IBA International) in the position corresponding to the off the cells during the irradiation procedure.

### Colony formation assay

For quantification of the clonogenic capacity, 50-1600 cells/well were plated in 6-well plates in a serial dilution of 1:2 (0 Gy: 50/100/200 cells/well; 2 Gy: 100/200/400 cells/well; 4 Gy: 200/400/800 cells/well; 6 Gy: 400/800/1600 cells/well). A day after plating, cells were irradiation with the respective beam at a doses of 0 Gy, 2 Gy, 4 Gy or 6 Gy. Immediately after irradiation, the medium was changed, and cells were incubated for 10 days (A172: 20 days) to allow colony formation from single cells. Afterwards, cells were fixed in 3.7% paraformaldehyde and 70% ethanol and subsequently stained with 0.05% Coomassie Brilliant Blue. Colonies (≥ 50 cells/colony) were counted under the microscope. Surviving fractions were calculated by determining the quotient of counted colonies to plated cells and subsequent normalization to the respective quotient for non-irradiated cells.

### Analysis of cell death

Analysis of cell death and apoptosis was performed 72 h after irradiation with respective beam at 0 Gy, 2 Gy or 5 Gy employing flow cytometry (FACS Calibur, Becton Dickinson, Heidelberg, Germany). To examine cell death by propidium iodide (PI purchased from Sigma-Aldrich, Deisenhofen, Germany) exclusion assay, cells were detached and stained with 10 µg/ml PI/PBS for 30 min in the dark. Subsequently, PI-positive (dead) cells were quantified by flow cytometry using channel FL-2. In addition, dissipation of mitochondrial membrane potential (ΔΨm) was analyzed using the ΔΨm-specific dye tetramethylrhodamine ethyl ester (TMRE, Molecular Probes/Thermo Fisher Scientific, Grand Island, NY, USA). After detachment, cells were stained in PBS containing 25 nM TMRE for 30 min before quantification by flow cytometry using channel FL-2. Apoptosis was quantified by analyzing DNA-fragmentation (sub-G1 cell fraction) after permeabilization and staining with PI. For this purpose, cells were incubated with a staining solution (50 μg/ml PI, 0.05% (v/v) Triton X-100, 0.1% (w/v) sodium citrate in PBS) for 30 min before analyzing PI fluorescence in channel FL-2.

### Analysis of CD9 and CD81 in glioblastoma cells

After detachment, cells were washed with PBS and counted using Neubauer cell chamber. 2x10^5^ cells were resuspended in 120 µL PBS and stained with 2 µL of each, PE-conjugated anti-human CD9 (EXBIO, Prague, Czech Republic) and FITC-conjugated anti-human CD81 (Beckman Coulter, Brea, CA, USA). 4 µL 7-AAD was added to identify dead cells. Compensation was performed in accordance with the standard procedure for flow cytometry using unstained and single-color dilutions of respective antibodies. Data was collected using a Cytoflex flow cytometer and Cytexpert 2.3 software (Beckman-Coulter).

### Quantification of extracellular vesicles by imaging flow cytometry IFCM analyses

After irradiation or sham-irradiation, the medium was discarded and replaced by RPMI-1640 medium supplemented with 10% of EV-depleted FBS (Invitrogen/Thermo Fisher Scientific, Waltham, MA, USA). After 72 h, the conditioned medium was centrifuged at 500x g for 5 min to eliminate cells. The supernatant was subjected to another centrifugation step at 2,000 x g for 15 min and filtrated using 0.22 µm filter (Sartorius, Göttingen, Germany). 10 µL of an antibody mix containing 0.5 µL PE-conjugated anti-human CD9 (EXBIO) and 0.5 µL FITC-conjugated anti-human CD81 (Beckman Coulter) were added to 90 µl of the filtrate. Unstained samples or dilutions of single-color stained controls of respective antibodies were used as controls according to the recommendations by the MIFlowCyt-EV framework (25). After incubation for 1 h at room temperature, samples were analyzed with an ImageStreamX Mark II instrument (Amnis/Luminex, Seattle, WA,USA) in duplicates with 5 minutes of acquisition time per well. Data was acquired at 60x magnification, low flow rate and deactivated “removed beads” option. Data analysis was performed using IDEAS software version 6.2 as previously described (22, 23). All fluorescent events were plotted against the side scatter. To improve the detection of fluorescent images, combined mask feature was used. Images were analyzed for coincidences (swarm detection) using the spot counting feature. Events with multiple spots were excluded from further analysis. All remaining events with low SSC (<500) and a fluorescent intensity higher than 300 were included in the calculation of concentrations. Gating strategy was provided in **Supplementary Figure S1**.

### Analysis of patient data

Expression and survival analysis of patients with glioblastoma multiforme (GBM) and low-grade glioma (LGG) were performed using data accessible at the GEPIA platform (http://gepia.cancer-pku.cn/). Median expression of CD9 and CD81 in GBM and LGG tumors was compared to that in non-malignant brain tissue. To estimate the impact of CD9 and CD81 expression on survival, patients with GBM and LGG were divided into two groups according to the median expression of the respective gene. Overall survival of low expressing cohort was compared to respective high expressing cohort.

### Statistics

Data represent mean values of at least three independent experiments ± standard deviation (SD). The results were subjected to statistical analysis using GraphPad Prism software (GraphPad Software, San Diego, CA, USA). Statistical significance was calculated using ANOVA followed by Bonferroni *post-hoc* test. P-value <0.05 was considered significant.

## Results

### Irradiation with high LET protons decreased clonogenic potential

**Figure 1:**
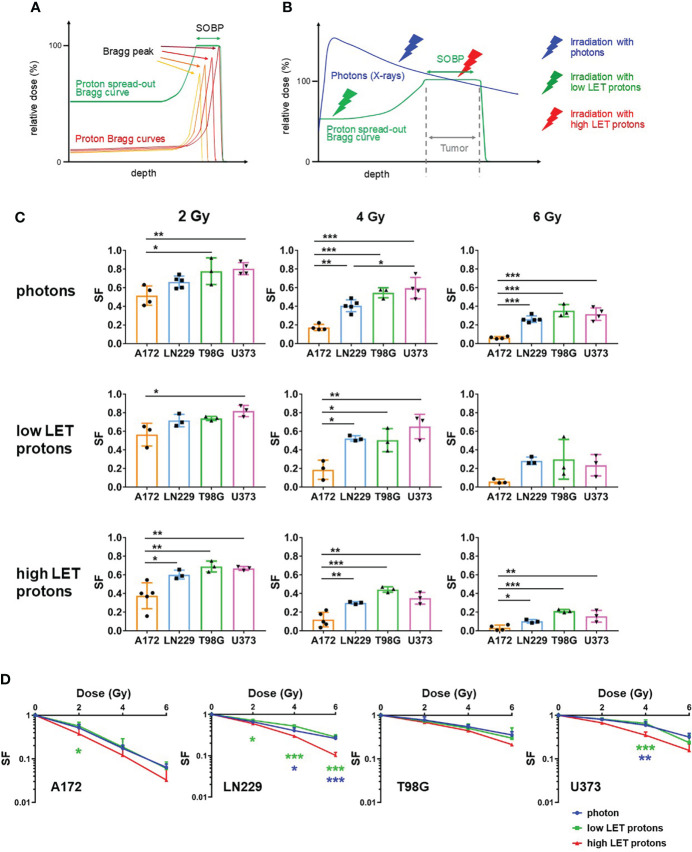
Irradiation with high LET protons reduced clonogenic survival more effectively than irradiation with photons or low LET protons. **(A)** Schematic presentation of a proton curve with spread out Bragg peak (SOBP) (green) built up of four proton beams with varying energies (low to high energy proton beams: dark red to orange). **(B)** Schema presenting dose deposition in tissue after irradiation with photons (blue) and protons (green). The thunderbolts indicate the region of the respective beams used to irradiate glioblastoma cells (blue: photons/X-rays; green: low LET protons; red: high LET protons). **(C, D)** Glioblastoma cells (A172, LN229, T98G and U373) were irradiated with respective beams at 0 Gy, 2 Gy, 4 Gy and 6 Gy and incubated to allow formation of colonies. Surviving fractions were calculated by determining the quotient of counted colonies to plated cells and subsequent normalization to the respective quotient for non-irradiated cells. **(C)** Surviving fraction (SF) of different cell lines was compared after irradiation with respective beams at 2 Gy (left panels), 4 Gy (middle panels) and 6 Gy (right panels). **(D)** The effect of the different beams (blue: irradiation with photons; green: irradiation with low LET protons; red: irradiation with high LET protons) on survival fraction was compared. N ≥ 3. *p < 0.05; **p < 0.01; ***p < 0.001 (blue: significance between irradiation with photons and high LET protons; green: significance between irradiation with low LET protons and high LET protons).

To irradiated glioblastoma cells with protons, we generated SOBP curve by overlaying protons Bragg curves of six different energies. A schematic generation of SOBP curves is pictured in **Figure 1A**. We irradiated glioblastoma cells with photons, low LET protons before SOBP and high LET protons at SOBP (**Figure 1B**). We analyzed radiosensitivity of glioblastoma cells (A172, LN229, T98G and U373) in response to photon-based and proton-based radiotherapy employing a clonogenic assay. **Figure 1A** displays the interaction of a proton beam with the tissue. The schema also presents the generation of the SOBP from single Bragg curves. A comparison of dose deposition in tumor and surrounding normal tissue after irradiation with photons and protons is visualized in **Figure 1B**. To assess clonogenic capacity, we irradiated glioblastoma cells with photons as well as with high LET or low LET protons at the respective dose to compare the effects of different irradiation beams. Of all cells, A172 cells reacted most sensitive in response to irradiation with photons, high LET or low LET protons (**Figure 1C**). Compared to A172 cells, LN229, T98G and U373 cells always displayed a higher surviving fraction (SF) after radiotherapy.

Comparing the response to photon-based irradiation with high LET and low LET proton-based irradiation, cells of all glioblastoma cell lines reacted more sensitive to irradiation with high LET protons than to irradiation with photons or low LET protons (**Figure 1D**). The effect was insignificant in T98G cells, while significantly decreased clonogenic potential after irradiation with high LET protons was detected in A172, LN229 and U373 cells. Moreover, our experiments demonstrate that irradiation with photons and low LET protons reduced the surviving fraction with comparable efficiency.

### Photon- and proton-based irradiation induced short-term toxicity with similar efficiency

**Figure 2:**
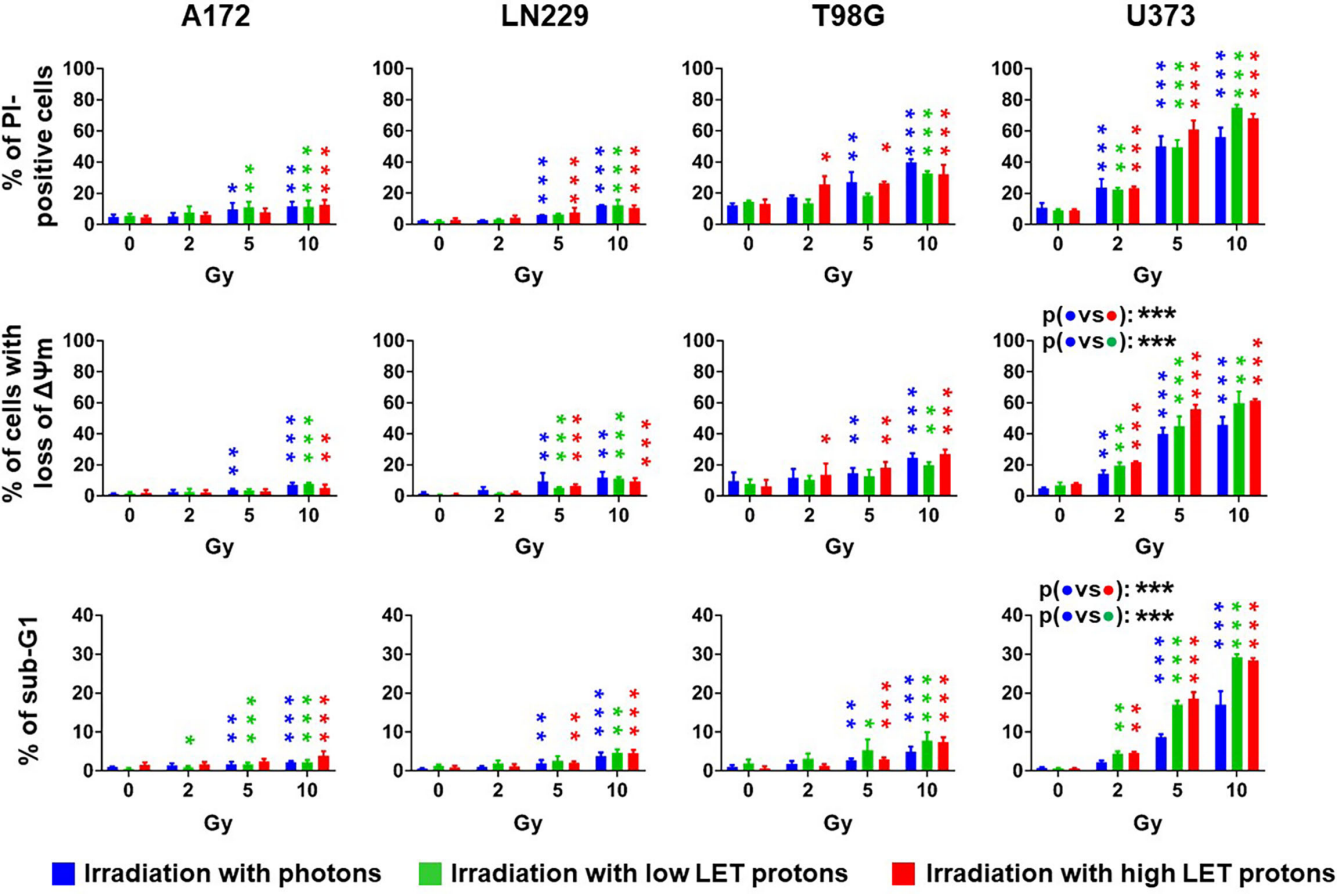
The irradiation technique does not affect radiation-induced cell death. Glioblastoma cells were irradiated with photons (blue), low LET protons (green) or high LET protons (red) at 0 Gy, 2 Gy, 5 Gy, and 10 Gy. 72 h later, cells death was determined by flow cytometry using propidium-iodide (PI) exclusion assay (upper panels) or by measuring dissipation of mitochondrial membrane potential (ΔΨm, middle panels). Apoptosis was determined by analyzing DNA fragmentation (cells with sub G1 fraction) after permeabilizing and staining cells with PI (lower panels). N ≥ 3. *, ** and *** above the bars indicate significance to respective sham-irradiated controls. *p < 0.05; **p < 0.01; ***p < 0.001.

Next, we assessed short-term cytotoxicity 72 h after irradiation with photons as well as high LET or low LET protons by flow cytometry. We determined cell death using propidium iodide (PI) exclusion assay (**Figure 2**, upper panels) and dissipation of mitochondrial membrane potential (ΔΨm) after staining cells with TMRE (**Figure 2**, central panels). Ionizing radiation induced cell death in a dose-dependent manner. A172 and LN229 cells reacted most refractory to radiation-induced cell death with less than 15% dead cells 72 h after irradiation with 10 Gy. U373 cells were the most sensitive cells of all four glioblastoma cell lines with more than 40% of dead cells after irradiation with 5 Gy, and more than 50% after irradiation with 10 Gy. After irradiation, cell death induction in T98G cells was more efficient than in A172 and LN229 cells, but less efficient than in U373 cells. Analysis of DNA fragmentation indicates that apoptosis substantially contributed to cell death in irradiated U373 cells, while apoptosis remained below 10% in any other cell line after irradiation with 10 Gy (**Figure 2**, lower panels). We did not detect any significant differences between irradiation with different beams. Only in U373 cells, irradiation with photons resulted in significantly less cell death and apoptosis than irradiation with high LET protons.

### Elevated expression of specific tetraspanin genes in low-grade glioma and glioblastoma correlated with shorter patient survival

A recent publication described an altered sEV profile in the blood plasma of patients with glioblastoma compared to healthy controls (15). These sEVs were further characterized by detecting the tetraspanin proteins CD9, CD81 and CD63. Since we barely detected CD63-positive sEVs in supernatants of our glioblastoma cells in preliminary experiments, we focused our examinations on the expression of CD9 and CD81 in human glioma tissues using accessible online data and in the different glioblastoma cells.

**Figure 3:**
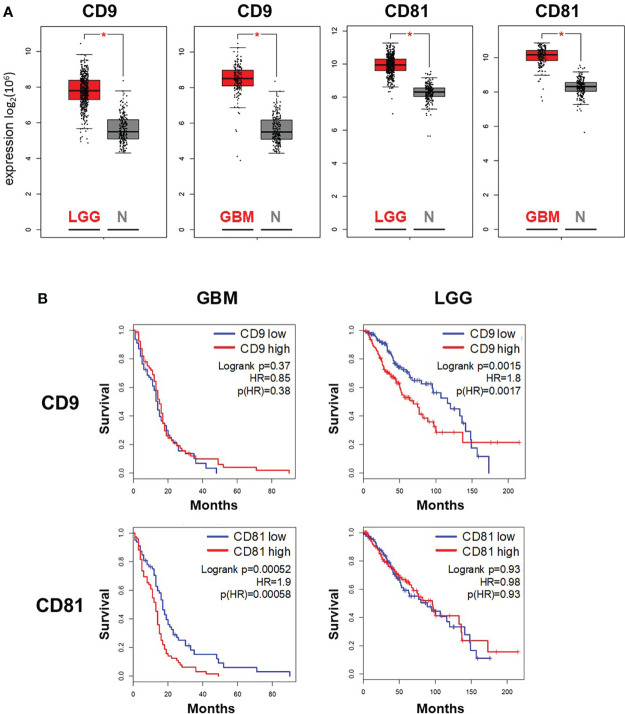
Expression of the tetraspanins CD9 and CD81 in glioma tumor samples correlates with survival of patients with glioma and glioblastoma. Expression **(A)** and survival analysis **(B)** of patients with glioblastoma multiforme (GBM) and low-grade glioma (LGG) were performed using data accessible at the GEPIA platform (http://gepia.cancer-pku.cn/). **(A)** Box plot analysis of CD9 and CD81 expression in GBM (n = 163) and LGG tissue (n = 518) in comparison to in non-malignant brain tissue (normal tissue N, n = 207)). * indicates significance (p < 0.05). **(B)** High expression of CD9 correlated with shorter survival in patients with LGG, while high expression of CD81 correlated with shorter survival in patients with GBM.

To analyze the importance of CD9 and CD81 expression for patients with glioma, we compared gene expression of both tetraspanin genes in glioblastoma (GBM. n = 167) and low-grade glioma (LGG, n = 518) probes with non-malignant brain tissue probes (n = 207) using GEPIA platform. Gene expression of both marker was significantly elevated in GBM as well as LGG samples compared to non-malignant samples (**Figure 3A**), suggesting that expression on CD9 and CD81 was elevated early during cancer progression to glioblastoma. In addition, we used the expression data at the GEPIA platform to predict survival depending on CD9 or CD81 expression. To this end, we divided patients with GBM or LGG in a high and low expressing group according to the median respective gene expression (GBM: n = 81 in each group, LGG: n = 257 in each group). Increased expression of CD9 correlated with shorter survival in patients with LGG, while no correlation between CD9 expression and survival was detected for patients with GBM (**Figure 3B**, upper panel). In contrast, high CD81 expression correlated with shorter survival of patients with high-grade GBM but not in patients with LGG (**Figure 3B**, upper panel). The data suggests that, depending on the WHO grade of glial tumors, expression levels of specific tetraspanin genes can predict patient survival.

### Glioblastoma cells released CD9 and CD81-positive EVs into the supernatant

**Figure 4:**
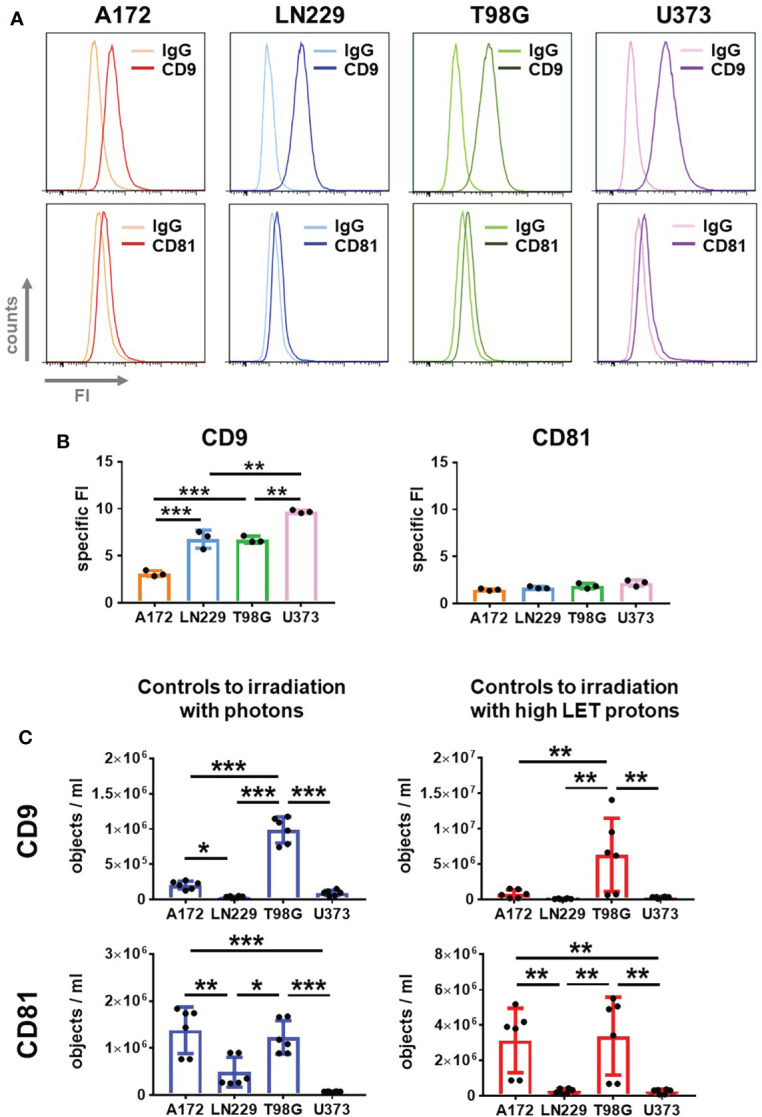
Glioblastoma cells secrete CD9 and CD81-positive EVs. **(A, B)** A172, LN229, T98G and U373 glioblastoma cells were permeabilized and stained with fluorophore-coupled antibody against CD9 or CD81 before analyzing by flow cytometry. As control, idiotype-matched antibodies (IgG) were used. Representative histograms showing fluorescence intensity (FI) are presented in **(A)**, while specific FI was calculated in **(B)**. N=3, **p < 0.01; ***p < 0.001. **(C)** 24 h after plating glioblastoma cells, medium was replaced by 2 mL of EV-free culture medium. Supernatant was collected after 72 h EVs in supernatant were quantified using IFCM analyses. Objects numbers in supernatants used as non-irradiated controls in irradiation experiments with photons (blue bars) and protons (red bars) were compared. N = 6. *p < 0.05; **p < 0.01; ***p < 0.001.

Next, we employed flow cytometry to analyze CD9 and CD81 protein levels in cells of the four glioblastoma cell lines. A clear CD9 signal was detected in all four glioblastoma cell lines (**Figure 4A**, upper panels). While similar CD9 signal intensities were detected in LN229 and T98G cells, CD9 signal intensities were lower in A172 cells. The highest CD9 signal intensities were detected in U373 cells (**Figure 4B**, left panel). In contrast, only weak CD81 signals were detected in all glioblastoma cells and did not differ between the cell lines (**Figure 4A**, lower panels and 4B right panel).

These results lead to the question whether the different CD9 and CD81 levels on the glioblastoma cells reflect the amount of CD9 and CD81-positive EVs secreted by these cells.

To address this question, we employed IFCM for single EV analysis in the supernatant of glioblastoma cells 72 h after medium change (**Figure 4C**). Moreover, we used the supernatant of non-irradiated cells acting as controls for irradiation with photons or high LET protons to compare experimental setups. The absolute numbers of CD9 and CD81-positive vesicles were always higher in the supernatant serving as controls to proton-based irradiation than in those serving as controls to photon-based irradiation. In both cases, however, T98G cells secreted the highest amount of CD9 and CD81-positive EVs. A172 cells secreted a similar amount of CD81-positive EVs as T98G cells, but fewer CD9-positive EVs than T98G cells, while LN229 and U373 cells secreted the lowest amount of CD9 and CD81-positive EVs. This data indicates that, depending on the experimental setup, the basal secretion of EVs by non-irradiated cells can be affected.

Our experiments indicate that the amount of secreted CD9 or CD81-positive EVs did not correlate with the tetraspanin levels in glioblastoma cells. We additionally concluded that the concentration of CD9 and CD81-positive EVs did not correlate with ionizing radiation-induced toxicity.

### Ionizing radiation increased secretion of extracellular vesicles in glioblastoma cells undergoing cell death

**Figure 5:**
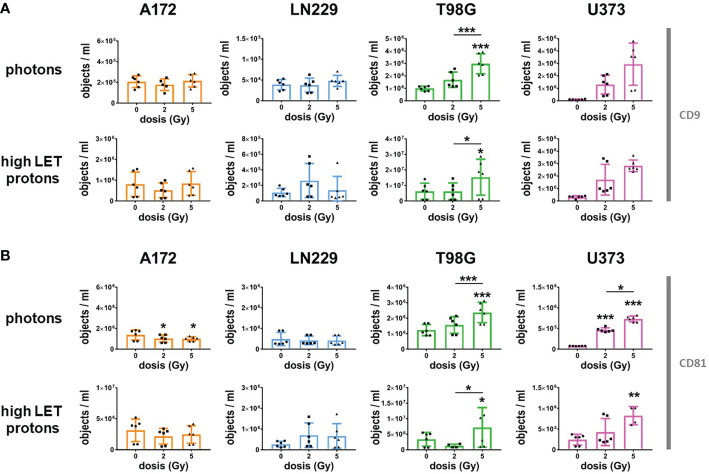
Radiation increased secretion of CD9 and CD81-positive EVs in T98G and U373 cells. 24 h after plating, glioblastoma cells were irradiated with photons or high protons at 0 Gy, 2 Gy or 5 Gy. Directly after irradiation, medium was replaced by 2 mL of EV-free culture medium. Supernatant was collected 72 h after irradiation and number of CD9- **(A)** or CD81-positive **(B)** objects was determined by using IFCM analyses. N = 4-6. *, ** and *** above the bars indicate significance to respective non-irradiated controls until otherwise indicated. *p < 0.05; **p < 0.01; ***p < 0.001.

Although EVs secreted by non-irradiated glioblastoma cells could not predict radiosensitivity, they might be helpful to monitor the response to radiotherapy. Therefore, we analyzed CD9 and CD81-positive EVs in the supernatant of glioblastoma cells 72 h after irradiation with photons or high LET protons, and compared the results to respective non-irradiated controls (**Figure 5**). We detected a dose-dependent, significant increase in secreted EVs following irradiation of T98G and U373 cells with photons and protons, while hardly any change in EV the amount of EVs was observed in the supernatants of irradiated A172 and LN229 cells. In contrast, the secretion of EVs secreted by A172 cells was even slightly lower after irradiation. Obviously, only GBM cells responding to ionizing radiation with cell death induction, particularly apoptosis, increased secretion of CD9 and CD81-positive EVs following irradiation. We concluded that the amount of secreted CD9 and CD81-positive EVs correlated with cell death induction in glioblastoma cells 72 h after irradiation.

## Discussion

Irradiation with protons allows confined deposition of energy at the SOBP plateau of the beam, targeting particularly the tumor while better sparing normal tissue (13). First trials comparing the effects of innovative proton-based with conventional photon-based radiotherapy in patients with glioblastoma demonstrated reduced cytotoxicity after irradiation with protons (26, 27). However, one study indicated that progression-free survival was not significantly increased (11). The biological response of glioblastoma cells to different modalities of radiotherapy, particularly to different beam qualities, has not been investigated yet. We aimed to compare the effect of photon-based and proton-based radiotherapy on GBM cell survival. Moreover, we characterized and analyzed EVs released by GBM cells, to predict radiosensitivity and to monitor the response to radiotherapy.

Our results revealed that GBM cells reacted more sensitive to irradiation with high LET protons, while radiation-induced cell death was not affected by the beam quality. Furthermore, we demonstrated that GBM cells released CD9 and CD81-positive EVs. The amount of released EVs could not predict radiosensitivity but increased after irradiation when GBM cells became apoptotic. We think that the increased release of CD9 and CD81-positive EVs might provide a promising marker to monitor GBM tumor response to radiotherapy.

### Effects of proton- and photon-based radiotherapy on glioblastoma cells

Using clonogenic assays, we demonstrated an increased sensitivity of glioblastoma cells to irradiation with protons in the SOBP plateau region (high LET protons) than to irradiation with photons or protons at SOBP entrance (low LET protons). An improved cytotoxicity after irradiation with low energy protons compared to photons was already described for other tumor entities including lung cancer, head and neck cancers and glioblastoma (26, 28, 29). We detected the improved response to irradiation with high LET protons notably in long-term experiments, measuring survival of cells able to proliferate and form colonies. In this context, a recent publication described the beneficial effect of proton-irradiation on glioma stem-like cells, emphasizing the anti-tumorigenic effect by lowering the clonogenic potential (30). Moreover, irradiation with protons causes more clustered DNA lesions than irradiation with photons (14). These proton-induced DNA lesions might be more difficult to repair than photon-based lesions and subsequently facilitate DNA damage-induced genetic and chromosomal alterations resulting in mitotic catastrophe or cell death (31). These effects might further decrease survival of clonogenic glioblastoma cells.

In addition, recent investigations suggested that tumor cells react more sensitive to proton beams than normal cells because of the repair defects (28, 32, 33). These repair defects resulted also in increased sensitivity to proton irradiation compared to photon irradiation. This is of special importance for GBM tumors, since altered DNA repair and chromosomal abnormalities are common features of GBM cells (34, 35). In summary, our data suggest that, in addition to sparing normal tissue, patients with glioblastoma might also benefit from increased cytotoxicity of proton-based radiotherapy.

Despite the improved cytotoxicity in long-term clonogenic assays after irradiation with high LET protons when compared to irradiation with photons, radiation-induced cell death after 72 h was not affected by the irradiation technique.

Indeed, long-term cytotoxicity measured in colony formation assay and short-term cytotoxicity measured by flow cytometry did not correspond. A172 cells reacting most sensitive to ionizing radiation in clonogenic assays hardly induced cell death. Eradication of clonogenic cells, however, does not require cell death induction. Alternatively, radiation-induced cell death can occur delayed in time, when initial DNA damage could not be repaired (31, 36). Furthermore, ionizing radiation is able to induced senescent phenotype associated with an irreversible cell cycle arrest (37, 38). Both, the delayed cell death as well as irreversible cell cycle arrest, prevent formation of colonies and therefore affect the outcome of long-term clonogenic assays but not the cell death 72 h after irradiation. Senescence together with cell death induction contributes to local tumor control by antagonizing tumor growth. On the other hand, senescent cells often develop a chronic secretory phenotype that rather promotes tumorigenesis and facilitates tumor regrowth after therapy (38, 39). Thus, therapy-induced cell death is preferable over senescence induction for a curative approach and long-lasting tumor control.

However, compared to low LET beams, irradiation with high LET beams could not improve cell death induction. Glioblastoma cells resistant to cell death induction by photon-based radiotherapy reacted also refractory to cell death induction by proton-based radiotherapy. Only U373 cells reacted more sensitive to irradiation with high LET protons than to irradiation with photons, suggesting that cell death induction might be enhanced in response to high LET beams in glioblastoma cells in some cells.

Therefore, based on our data, we concluded that proton-based radiotherapy is an alternative treatment option to conventional radiotherapy for patients with glioblastoma.

### Extracellular vesicles are potential markers monitoring tumor response

Regardless of the exact mechanism induced by ionizing radiation, an easy-to-handle marker predicting the response to radiotherapy would be desirable. A recent publication described in this context glioblastoma-derived EVs containing CD9 and CD81 in blood samples (15). CD9 and CD81 are cell surface glycoproteins with four transmembrane domains belonging to the tetraspanin family. Tetraspanins are proposed to form complex protein networks in biological membranes by recruiting other partner proteins into the tetraspanin-enriched microdomains, thus regulating signaling and cell response to extracellular signals (40). Increased expression of CD9 and CD81 was detected in multiple tumors and associated with increased tumor cell motility and improved tumor growth (41–43). In glioblastoma, increased CD9 expression was associated with maintenance of glioblastoma stem-like cells (43, 44), while CD81 was associated with enhanced resistance to radiotherapy by promoting nuclear translocation of RAD51, a protein involved in detection and repair of DNA double strand breaks (45). Analyzing data accessible at the GEPIA platform, we detected increased expression of CD9 and CD81 in glioblastoma and low-grade glioma, proposing an upregulation of both tetraspanins early in the tumorigenesis of gliomas. Moreover, in patients with glioblastoma, high CD81 expression correlated with shorter survival, while, in patients with low-grade glioma, high CD9 expression correlated with shorter survival. Thus, the data suggest a switch in pro-survival signaling through tetraspanins during glioma tumorigenesis, which is regulated on the post-translational level. It is possible that the upregulation of CD9 expression early in the glioma genesis facilitates accumulation of radioresistant stem-like cells later on (46). The glioblastoma stem-like cells constitutively up-regulate DNA damage repair as response to changed environmental conditions (47). In this manner, CD81-mediated DNA repair later in the genesis of glioma might become more important for tumor cell survival. As a protein regulating complex assembly at the membrane, CD81 also coordinates the transport of its interacting partners to vesicles, thus governing the compositions of EVs (48). After EV uptake, the surrounding normal and tumor cells might change their behavior creating a more favorable environment for glioblastoma cells and increasing therapy resistance (49, 50). That way, CD81 might indirectly affect the therapeutic response of glioblastoma cells.

Moreover, the EV content could provide us not only with information about the tumor generating the EVs, but could also allow a deduction how cells behave after EV uptake. Based on these observations, we concluded that specific proteins on extracellular vesicles such as CD9 and CD81 are excellent markers to monitor behavior of glioblastoma cells. We detected varying concentrations of CD9 or CD81-positive EVs in the supernatant of different glioblastoma cell lines, but could not use this data to predict the response to radiotherapy. The number of CD9 or CD81-positive vesicles in supernatant of non-irradiated glioblastoma did not correlate with radiation-induced cytotoxicity in long-term or short-term assays. After irradiation, however, the amount of CD9 and CD81-positive EVs increased in supernatant when glioblastoma cells underwent cell death, particularly apoptosis. This effect was similar after irradiation with high LET protons and photons. We therefore concluded that the changed secretion of CD9 and CD81-positive EVs can be used to monitor glioblastoma cell response to radiotherapy irrespective of the radiation technique.

Analyzing DNA fragmentation, we concluded that apoptosis contributed to cell death induction after irradiation. DNA might be fragmented without activation of caspases, the main proteases activated during apoptosis (51). We did not analyze further apoptotic markers in this experimental setup. Our previous analyses demonstrated however caspase activation in U373 cells and a weaker in T98G cells after irradiation with photons (52). In analogy to the results obtained after irradiation with photons, we think it very likely that proton-based irradiation also induced apoptosis in T98G cells when irradiated at a doses of 5 Gy or higher.

A previous study demonstrated that vesicles released by glioblastoma cells undergoing apoptosis could promote malignancy in the surviving cells after uptake of these EVs (53). We did not analyze EV uptake in the different glioblastoma cells or the behavior of glioblastoma cells after re-irradiation, thus we have no data to support this observation. So far, our results suggest that the release of EVs is rather a response of glioblastoma cells undergoing radiation-induced cell death. It also remains unclear whether the content of released EVs from cell undergoing apoptosis in response to radiotherapy is the same as from cell undergoing apoptosis in response to other stimuli.

Surprisingly, the absolute numbers of extracellular vesicles in supernatants serving as respective controls to irradiation with photons or protons differed. Compared to irradiation with photons, the less standardized procedure for irradiation with protons is also reflected by higher error bars. The slightly different handling of cells during irradiation, such as the prolonged time at room temperature during irradiation with protons, might have induced additional stress to the cells and increase EV release. Increased EV release in response to cell stress induced by hypoxia, nutrient deprivation, heat stress and oxidative stress was previously reported (49, 54). The environmental conditions might additionally affect EV release from glioblastoma cells after irradiation.

Despite many unresolved questions that we were not able to address so far, our data suggest the changed release of CD9 and CD81-positive EVs by glioblastoma as marker to monitor glioblastoma response to radiotherapy. The detection of these vesicles could be an alternative to non-invasive detection techniques requiring expensive equipment and trained staff.

## Data availability statement

The original contributions presented in the study are included in the article/**Supplementary Material**. Further inquiries can be directed to the corresponding author.

## Author contributions

JR designed the study and supervised the work. SJ, supported by MP, performed most of the experiments and analyzed the data. TT measured EVs and analyzed the data. VJ helped with data interpretation. BK and MV performed the dosimetry and irradiation with protons. BT provided access and experimental time at the proton facility. SJ coordinated the experiments in the different departments. BG, TT, and BT advised on the analysis of extracellular vesicles. SJ, BG and JR drafted the manuscript. All authors revised the manuscript and gave their final approval.

## Acknowledgments

We thank Angelika Warda for technical support and George Iliakis for providing access to irradiation facility (irradiation with photons). The work was supported by grants of the Deutsche Krebshilfe/Mildred-Scheel-Stiftung (70112711) to VR and JR and of the German Research Training Group GRK1739/2 to VJ.

## Conflict of interest

The authors declare that the research was conducted in the absence of any commercial or financial relationships that could be construed as a potential conflict of interest.

## Publisher’s note

All claims expressed in this article are solely those of the authors and do not necessarily represent those of their affiliated organizations, or those of the publisher, the editors and the reviewers. Any product that may be evaluated in this article, or claim that may be made by its manufacturer, is not guaranteed or endorsed by the publisher.
