# Supplementary material for: CD9- and CD81-positive extracellular vesicles provide a marker to monitor glioblastoma cell response to photon-based and proton-based radiotherapy
Source: Front Oncol. 2022 Sep 20;12:947439. doi: 10.3389/fonc.2022.947439 (PMC9530604; doi:10.3389/fonc.2022.947439)
Supplement: Supplementary file 1 [file DataSheet_1.docx]

**Figure S1: Gating strategy to detect CD9 and CD81-positive extracellular vesicles**


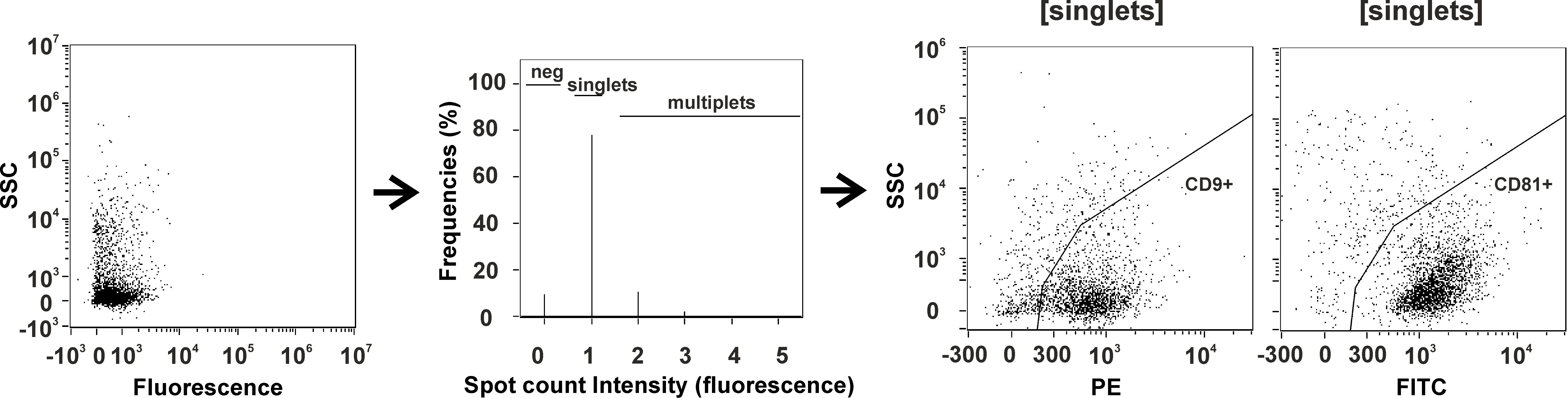


After 72 h of cell cultivation, the conditioned medium was collected and centrifuged at 900x g for 5 min to eliminate cells. The supernatant was subjected to another centrifugation step at 2,000 x g for 15 min and filtrated using 0.22 µm filter (Sartorius, Göttingen, Germany). An antibody mix containing 0 PE-conjugated anti-human CD9 (EXBIO) and FITC-conjugated anti-human CD81 were added to the filtrate. Unstained samples or dilutions of single-color stained controls of respective antibodies were used as controls according to the recommandations by the MIFlowCyt-EV framework. Samples were analyzed with an ImageStreamX Mark II instrument (Amnis/Luminex, Seattle, WA,USA) in duplicates with 5 minutes of acquisition time per well. Data was acquired at 60x magnification, low flow rate and deactivated “removed beads” option. Data analysis was performed using IDEAS software version 6.2. All fluorescent events were plotted against the side scatter (left panel). To improve the detection of fluorescent images, combined mask feature was used. Images were analyzed for coincidences (swarm detection) using the spot counting feature (middle panel). Events with multiple spots were excluded from further analysis. All remaining events with low SSC (<500) and a fluorescent intensity higher than 300 were included in the calculation of concentrations (right panels).
